# Sgt1 Regulates α-Synuclein Subcellular Localization and Expression of Parkinson’s Disease Related Genes, *PINK1* and *PARK9*

**DOI:** 10.3390/biom11111675

**Published:** 2021-11-11

**Authors:** Anastasiia Bohush, Agnieszka Góral, Małgorzata Sierant, Barbara Nawrot, Wiesława Leśniak, Anna Filipek

**Affiliations:** 1 Nencki Institute of Experimental Biology, Polish Academy of Sciences, 02-093 Warsaw, Poland; a.bohush@nencki.edu.pl (A.B.); agnieszkabozena.goral@gmail.com (A.G.); w.lesniak@nencki.edu.pl (W.L.); 2 Centre of Molecular and Macromolecular Studies, Polish Academy of Sciences, 90-363 Lodz, Poland; msierant@cbmm.lodz.pl (M.S.); bnawrot@cbmm.lodz.pl (B.N.)

**Keywords:** α-synuclein, SGT1, subcellular localization, Parkinson’s disease, *PINK1*, *PARK9*

## Abstract

The SGT1 protein is highly expressed in the mammalian brain, particularly in neurons of the hippocampus and cortex, and in Purkinje cells of the cerebellum. There are literature data indicating that the protein may be involved in pathogenesis of neurodegenerative disorders such as Parkinson’s disease (PD). In the present work we have found that SGT1 protected cells from the toxicity of rotenone, an agent that evokes behavioral and histopathological symptoms of PD. To gain more insight into the possible mechanism underlying the protective action of SGT1 we looked at α-synuclein subcellular distribution in HEK293 cells with an altered SGT1 level. By immunofluorescent staining we have found that in HEK293 cells overexpressing SGT1 α-synuclein was mainly localized in the cytoplasm while in control cells it was present in the nucleus. Accordingly, when SGT1 expression was silenced, α-synuclein was predominantly present in the nucleus. These results were then confirmed by subcellular fractionation and Western blot analysis. Moreover, we have found that altered level of SGT1 in HEK293 cells influenced the expression of PD related genes, *PINK1* and *PARK9.* Altogether, our results point to SGT1 as an important factor that might be involved in the pathogenesis of Parkinson’s disease (PD).

## 1. Introduction

Parkinson’s disease (PD) is a progressive neurodegenerative disorder characterized by the presence of characteristic intracellular inclusions, called Lewy bodies (LBs), composed of misfolded α-synuclein, and by selective loss of dopaminergic neurons in substantia nigra. The majority of PD cases are sporadic in origin. However, mutations in several proteins, including α-synuclein, PTEN-induced kinase 1 (PINK1) or Polyamine-Transporting ATPase 13A2 (encoded by *PARK9* gene), have been linked to PD [1].

Previous studies have shown that under physiological conditions α-synuclein is involved in regulating membrane lipid content, vesicle trafficking, dopamine metabolism or SNARE-dependent complex assembly [2]. α-Synuclein is localized at presynaptic terminals, however, it is also found in the cytoplasm, mitochondria, endoplasmic reticulum and Golgi apparatus/lysosomes [3]. Nuclear localization of α-synuclein has been described in transgenic *Drosophila* [4], mice [5] and in cultured cells [6], but its physiological significance is not fully understood. Previously, it has been shown that nuclear α-synuclein promotes neurotoxicity by inhibiting histone acetylation, while sequestration of α-synuclein in the cytoplasm is protective [4]. Such effect can be due to the involvement of α-synuclein in regulation of transcription through binding to DNA [7], histones [8] and chromatin [9]. For instance, it has been shown that α-synuclein is involved in downregulation of Nurr1, a nuclear receptor essential for the development and specification of midbrain dopamine neurons [10]. Binding of α-synuclein to DNA and subsequent down-regulation of multiple cell cycle-related genes has been recently determined by chromatin immunoprecipitation assay (ChIP) followed by next-generation sequencing (ChIP-seq) in Lund human mesencephalic (LUHMES) cells and in transgenic mice overexpressing α-synuclein [7].

SGT1 (suppressor of G2 allele of Skp1) is a component of the CBF3 kinetochore and SCF ubiquitin ligase complexes [11]. Importantly, SGT1 binds Hsp90 and is a component of the chaperone complexes [12]. It can also play a role in innate immunity through stabilization of the NOD1 receptor [13]. SGT1 is highly expressed in neurons of the hippocampus and cortex and in Purkinje and glial cells of the white matter of the cerebellum [14]. Interestingly, SGT1 seems to be involved in several neurodegenerative disorders including Alzheimer’s disease (AD) [14], PD or dementia with Lewy bodies (DLB) [15]. In particular, in cortical regions of AD brain, the density of SGT1-stained neurons was lower compared to corresponding regions of control brain [14]. This finding implies that SGT1-immunopositive cells may be selectively affected in AD. In turn, SGT1 mRNA level was found to be higher in frontal and temporal cortex of PD and in substantia nigra of DLB brains. Interestingly, SGT1 was not found in Lewy bodies, which are composed mainly of aggregated/phosphorylated α-synuclein [15].

One of the cellular mechanisms that control α-synuclein folding involves chaperone proteins/heat shock proteins (HSPs). Numerous studies have shown that some chaperones and co-chaperones protect cells from α-synuclein aggregation and toxicity [16]. For instance, a novel chaperone/co-chaperone, CacyBP/SIP, inhibits α-synuclein aggregation in in vitro assays and protects cells from toxicity induced by rotenone, an agent that evokes behavioral and histopathological symptoms of PD [17]. Taking into account similar domain organization and high amino acid sequence homology between CacyBP/SIP and SGT1 [18], we hypothesized that SGT1 might also exert beneficial effects counteracting α-synuclein toxicity.

Results obtained in the present work have shown that SGT1 protects HEK293 cells from rotenone-induced toxicity and that it may be involved in regulating α-synuclein partitioning between nucleus and cytoplasm. Moreover, the results point that altered SGT1 level has an effect on the expression of PD-related genes such as *PINK1* and *PARK9.*

## 2. Materials and Methods

### 2.1. Plasmids

List of plasmids used in this work is shown in Table 1. p3×FLAG-CMV-10-SGT1B was prepared as follows: a DNA fragment containing the coding sequence of human SGT1B was amplified by PCR using pDEST17 as a template plasmid (kindly provided by K. Kitagawa, Department of Molecular Medicine, Greehey Children’s Cancer Research Institute, San Antonio, TX, USA) and the following primers: forward 5′-TAATAAAGCTTATGGCGGCGGCTGCAGCA-3′ and reverse 5′-ACGCGCGAATTCTTAGTACTTTTTCCATCC-3′. The PCR product was digested with HindIII and EcoRI restriction enzymes (both from Thermo Fisher Scientific, Waltham, MA, USA) and introduced into the p3×FLAG-CMV-10 plasmid (Sigma-Aldrich, St. Louis, MO, USA) previously digested with the same enzymes. The correctness of the cloned DNA sequence was confirmed by sequencing (Institute of Biochemistry and Biophysics, Warsaw, Poland).

For silencing of SGT1 (A and B isoform) the sense: 5′-CACCGCACAGTATTATTGTCAAAGAGTGTGCTGTCCTCTTTGACAATAATACTGTGCTTTTT-3′ and antisense: 5′-GCATAAAAAGCACAG TATTATTGTCAAAGAGGACAGCACACTCTTTGACAATAATACTGTGC-3′ oligonucleotides comprising the target sequence 5′-GCACAGTATTATTGTCAAAGA-3′, corresponding to a fragment of *SGT1,* were synthetized, hybridized and the double strand product was cloned into the modified pPUR plasmid (Takara Bio Inc., Shiga, Japan), containing the U6 promoter for RNA Pol III, using BspMI restriction enzyme (Thermo Fisher Scientific, Waltham, MA, USA) [19] to obtain the pPURhU6-SGT1 plasmid. Correctness of the cloning was confirmed by sequencing as described above.

### 2.2. Cell Culture and Transfection

HEK293 cells (ATCC CRL-1573^TM^) were cultured in Dulbecco’s Modified Eagle Medium (DMEM, Sigma-Aldrich, St. Louis, MO, USA) containing 10% (*w*/*v*) fetal bovine serum (FBS) (Thermo Fisher Scientific, Waltham, MA, USA), penicillin (100 U/mL) and streptomycin (100 µg/mL) (both from Sigma-Aldrich, St. Louis, MO, USA) at 37 °C and under 5% CO_2_. Cells (75–80% confluent) were transfected with an appropriate plasmid using Lipofectamine2000 (Thermo Fisher Scientific, Waltham, MA, USA) according to manufacturer’s protocol. 

To obtain HEK293 cell line stably overexpressing SGT1 (A and B isoform), cells at 75% confluency were transfected with p3×FLAG-CMV-10-SGT1A and p3×FLAG-CMV-10-SGT1B plasmids or p3×FLAG-CMV-10 plasmid (control). After 24 h, cells were treated with geneticin (Thermo Fisher Scientific; Waltham, MA, USA) at a final concentration of 500 ng/mL. The morphology of cells was monitored every day under the light microscope (TMS, Nikon, Melville, NY, USA) and the medium, supplemented with geneticin, was changed every 2 days. Finally, cells were maintained in medium containing 250 ng/mL geneticin. HEK293 cells with overexpression of SGT1 (both isoforms) and control ones were transiently transfected with pcDNA4-α-synuclein-3×FLAG or pcDNA4-3×FLAG plasmid (control) as described above and analyzed 24 h later.

In order to silence SGT1 (A and B isoform), HEK293 cells were transiently transfected with pPURhU6-SGT1 plasmid (encoding shRNA against mRNA for SGT1) or pPURhU6-Luc4 plasmid (encoding shRNA against *Renilla* Luciferase mRNA) as a control, using Lipofectamine2000 as described above. Then, 24 h later, these cells were transfected with pcDNA4-α-synuclein-3×FLAG or pcDNA4-3×FLAG and examined after an additional 24 h.

### 2.3. Preparation of Protein Lysates

To obtain cytoplasmic and nuclear fractions, cells were rinsed with ice-cold PBS, (Sigma-Aldrich, St. Louis, MO, USA) collected and lysed according to the Nuclear and Cytoplasmic extraction kit procedure, NE-PER (Thermo Fisher Scientific, Waltham, MA, USA). To obtain total protein lysate cells were lysed in RIPA buffer (Millipore, Burlington, MA, USA), passed 20 times through syringe and incubated on ice for 30 min. Protein lysates were centrifuged at 12,000× *g* for 20 min at 4 °C. The supernatant was collected and protein concentration was measured by Bradford’s method (Bio-Rad, Hercules, CA, USA). A total of 30 μg of protein lysate was taken, precipitated in 1 mL ice-cold acetone and kept at −20 °C until use.

### 2.4. Western Blot Analysis

Proteins precipitated in ice-cold acetone were centrifuged at 20,000× *g* for 15 min at 4 °C, mixed with Laemmli’s sample buffer, incubated at 95 °C for 5 min and subjected to SDS-PAGE according to Laemmli [21]. SDS-PAGE and transfer onto nitrocellulose membrane were carried out as described by Góral et al. [22]. In order to avoid non-specific binding of antibodies, the nitrocellulose was incubated in 5% *w*/*v* lyophilized fat-free milk in TBS-T for 1 h at room temperature. Then, the membrane was incubated overnight at 4 °C with primary mouse anti-α-synuclein (Abcam; Cambridge, UK, cat no ab1903) or rabbit anti-SGT1 (BD Biosciences, San Jose, CA, USA) antibodies, both diluted 1:1000. Afterwards, the nitrocellulose was washed in TBS-T and allowed to react for 1 h in room temperature with secondary anti-mouse IgG (Jackson ImmunoResearch, West Grove, PA, USA) or anti-rabbit IgG (Millipore, Burlington, MA, USA) antibodies conjugated with horseradish peroxidase (HRP) diluted 1:10,000. The level of β-actin, detected by mouse monoclonal antibody conjugated with HRP (Sigma-Aldrich, St. Louis, MO, USA) diluted 1:10,000, was used as a reference of protein loading for total cell lysate and for cytoplasmic fraction. The level of PARP, detected by rabbit polyclonal antibody (Cell Signaling Technology, Danvers, MA, USA) diluted 1:1000, was used as a reference for the nuclear fraction. The intensities of protein bands on an X-ray film (Kodak, Rochester, NY, USA) were quantified using a manual gel and film documentation in an Ingenius densitometer (Syngene, Frederick, MD, USA) and the Gene Tools software (Syngene, Frederick, MD, USA). Statistical analysis was performed as described below (Section 2.9).

### 2.5. Immunofluorescence Staining and Confocal Microscopy

Cells were cultured on glass cover slips, fixed with 3% paraformaldehyde (Sigma-Aldrich, St. Louis, MO, USA) for 20 min at room temperature and washed three times for 3 min in PBS. Afterwards cells were incubated for 10 min in 10 mM PIPES, 25 mM HEPES, 10 mM EGTA, 4 mM MgCl_2_, pH 7.0 (ICCH buffer) containing 50 mM NH_4_Cl and washed again three times for 3 min with PBS. Coverslips were treated for 4 min at 4 °C with 0.1% Triton X-100 in ICCH buffer. In the subsequent step cells were incubated in 5% BSA solution (Sigma-Aldrich, St. Louis, MO, USA) for 1 h and then overnight at 4 °C with mouse anti-α-synuclein antibody (Abcam; Cambridge, UK, cat no ab1903), diluted 1:200, and rabbit anti-SGT1 antibody (BD Biosciences, San Jose, CA, USA), diluted 1:200. The reaction with secondary antibody was carried out at room temperature for 1.5 h using anti-rabbit IgG antibody conjugated with Alexa Fluor 488 or anti-mouse IgG antibody conjugated with Alexa Fluor 555 (both from Thermo Fisher Scientific, Waltham, MA, USA) diluted 1:500 in 2.5% BSA solution. After the final wash with PBS, the coverslips were mounted on slides with mounting media containing DAPI (VectaShield, Sigma-Aldrich, St. Louis, MO, USA). Immunofluorescence staining was analyzed under a confocal microscope (LSM 800, Carl Zeiss, Jena, Germany), equipped with a 63 × oil objective. 

### 2.6. Fluorescence-Based Quantification of Nuclear-Cytoplasmic Localization 

Quantifications were performed according to [23]. The intensity of fluorescent staining, which reflects α-synuclein localization, was calculated in selected region of interest (ROI) using the ImageJ 1.42q software (NIH, Bethesda, MD, USA) for both nucleus and cytoplasm. Then, the mean fluorescence value for nucleus and cytoplasm was calculated for 20 cells taken from three independent experiments. 

### 2.7. Real Time/Quantitative PCR (RT-qPCR)

Total RNA was isolated from HEK293 cells using the Extractme kit (Blirt DNA, Gdansk, Poland). RNA (2 μg) was then reverse-transcribed using M-MLV Reverse Transcriptase and random nonamers (Sigma–Aldrich, St.Louis, MO, USA). Relative mRNA levels were measured by RT-qPCR using the SYBRGreen system (Thermo Fisher Scientific, Waltham, MA, USA) with 18S rRNA as a standard. The primers used are listed in Table 2. The results were analyzed by absolute quantification with a relative standard curve and normalized to 18S rRNA using the comparative ΔΔCt method.

### 2.8. Cell Viability Assay

HEK293 cells, with stable overexpression of 3×FLAG-SGT1 (SGT1A and SGT1B) or 3×FLAG alone (control), were counted in an automatic cell counter (NanoEnTek, Guro-gu, Seoul, Korea) and seeded into wells of a 24-well plate. Then, cells were transfected with plasmid encoding α-synuclein, left for 24 h and transferred to a 96-well plate. After 7 h, the cells were treated with 5 μM rotenone (Sigma–Aldrich, St. Louis, MO, USA) while control cells treated with an equivalent volume of solvent (96% ethanol). Cells were left for 18 h and MTS assay (Promega, Madison, WI, USA) was performed according to manufacturer’s protocol. The level of formazan was measured by recording changes in the absorbance at 490 nm using a microplate reader (Tecan, Morrisville, NC, USA). Viability of cells overexpressing 3×FLAG-SGT1 (SGT1A and SGT1B) or 3×FLAG alone was compared to the viability of those treated with solvent alone.

### 2.9. Statistical Analysis

Experiments were performed at least in triplicates and results are presented as means ± SEM. Results obtained from analysis of fluorescence intensity, Western blots and RT-qPCR were analyzed by Student’s *t*-test using Microsoft Excel. Results of the MTS assay were analyzed by one-way ANOVA. The level of statistical significance was set at * p ≤ 0.05; ** p ≤ 0.01; *** p ≤ 0.001.

## 3. Results

### 3.1. SGT1 Protects HEK293 Cells from Rotenone-Induced Toxicity 

**Figure 1:**
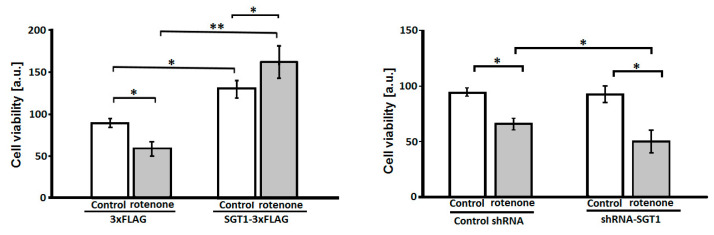
**Effect of altered SGT1 level on viability of HEK293 cells treated with rotenone.** (**Left panel**) Cells overexpressing SGT1-3×FLAG or 3×FLAG were treated with solvent (control, white bar) or rotenone (grey bar). (**Right panel**) Cells transfected with plasmid encoding shRNA against SGT1 mRNA or control shRNA were treated with solvent (control, white bar) or rotenone (grey bar). Cell viability was quantified using MTS assay. Statistical analysis was performed for results obtained from three independent experiments. * *p* ≤ 0.05; ** *p* ≤ 0.01.

**Figure 2:**
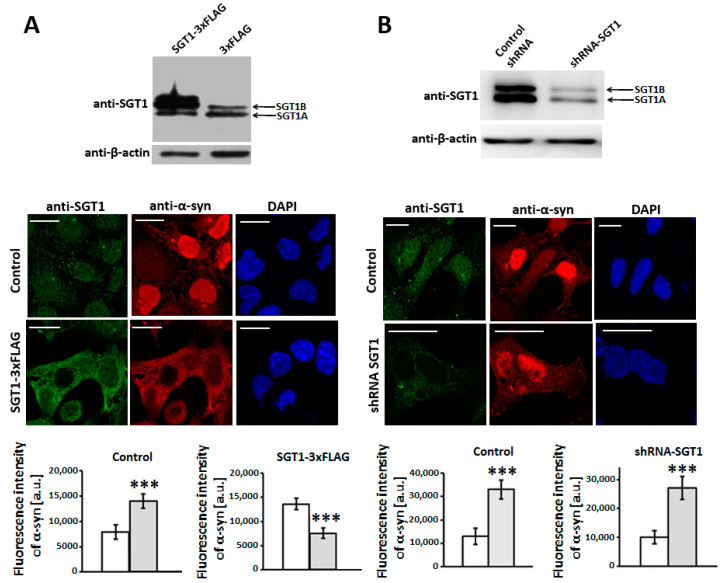
**Influence of altered level of SGT1 on α-synuclein subcellular localization in HEK293 cells.** (**A**,**B**, **upper panels**) Representative Western blots showing the level of SGT1 after overexpression (**A**) or silencing (**B**) of both isoforms (SGT1A and SGT1B) in total cell lysate. (**A**,**B**, **middle panels**) Immunofluorescence staining of cells overexpressing SGT1 (SGT1-3×FLAG) (**A**) or with diminished SGT1 level (shRNA-SGT1) (**B**) performed using anti-SGT1 and anti-α-synuclein antibodies. Nuclei, stained with DAPI, are in blue. Scale bar—20 μm. (**A**,**B**, **lower panels**) Statistical analysis of α-synuclein fluorescence staining in cytoplasm (white bar) and nucleus (grey bar) was performed for 20 cells. *** *p* ≤ 0.001.

At first, we checked whether SGT1, as a co-chaperone of Hsp90, has an effect on the viability of HEK293 cells treated with rotenone, an agent that evokes behavioral and histopathological symptoms similar to those observed in PD [24]. For that, the MTS assay was performed. As it can be seen in Figure 1, SGT1 overexpression (left panel) increases cell viability while silencing (right panel) has no effect. Moreover, overexpression of SGT1 protects HEK293 cells against rotenone-induced toxicity (Figure 1, left panel). On the other hand, rotenone-treated cells with silenced SGT1 were less viable than rotenone-treated control cells (Figure 1, right panel). The mean rates of SGT1 overexpression or silencing (shown in Figure 2A,B, upper panels) calculated from three different cell transfections were 71% +/− 11.3% and 49% +/− 5.5%, respectively.

### 3.2. SGT1 Affects α-Synuclein Subcellular Localization in HEK293 Cells

To get more insight into the possible mechanism underlying the protective action of SGT1 we looked at α-synuclein subcellular distribution in control HEK293 cells and in cells with altered level of SGT1 (Figure 2A,B, upper panels). For that we applied immunocytochemistry with the use of anti-SGT1 and anti-α-synuclein antibodies. It was found that in control cells α-synuclein was predominantly localized in the nucleus while in cells overexpressing SGT1, α-synuclein was mainly present in the cytoplasm (Figure 2A, middle panels) in which it could be detected in SGT1-containing complexes (Figure S1). In order to confirm this finding, we analyzed HEK293 cells with diminished level of SGT1. We found that in these cells α-synuclein was predominantly present in the nucleus (Figure 2B, middle panels). Statistical analysis of fluorescence intensities in the cytoplasmic and nuclear areas was performed in parallel and the results are shown in Figure 2A,B, lower panels.

**Figure 3:**
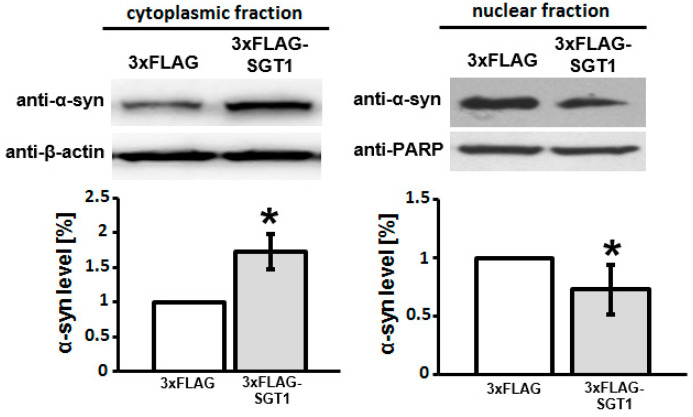
**Effect of increased SGT1 level on α-synuclein subcellular localization in HEK293 cells.** (**Upper panels**) Representative Western blots showing the level of α-synuclein in cytoplasmic and nuclear fractions obtained from cells overexpressing SGT1 (SGT1-3×FLAG) or control ones (3×FLAG). (**Lower panels**) Statistical analysis of densitometry results obtained from 3 independent experiments. * *p* ≤ 0.05.

To confirm the immunocytochemistry results, subcellular fractionation and Western blot analysis were performed. As it can be seen in Figure 3, overexpression of SGT1 leads to an increase in α-synuclein level in the cytoplasmic fraction and to a decrease in the level of this protein in the nuclear fraction when compared with control cells. 

**Figure 4:**
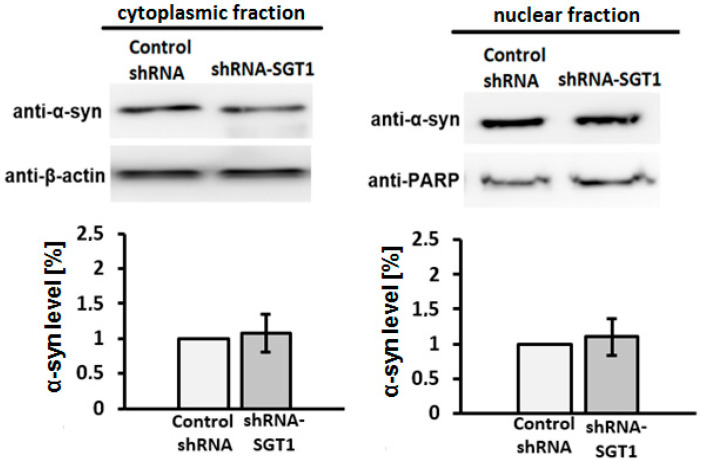
**Effect of diminished SGT1 level on α-synuclein subcellular localization in HEK293 cells.** (**Upper panels**) Representative Western blots showing the level of α-synuclein in cytoplasmic and nuclear fractions obtained from cells with diminished SGT1 level (shRNA-SGT1) or control ones (control shRNA). (**Lower panels**) Statistical analysis of densitometry results obtained from three independent experiments; no statistically significant differences were found.

Notably, silencing of SGT1 did not cause any statistically significant differences in α-synuclein protein level either in the cytoplasm or in the nucleus (Figure 4).

### 3.3. Altered SGT1 Level Triggers Changes in PINK1 and PARK9 Gene Expression

**Figure 5:**
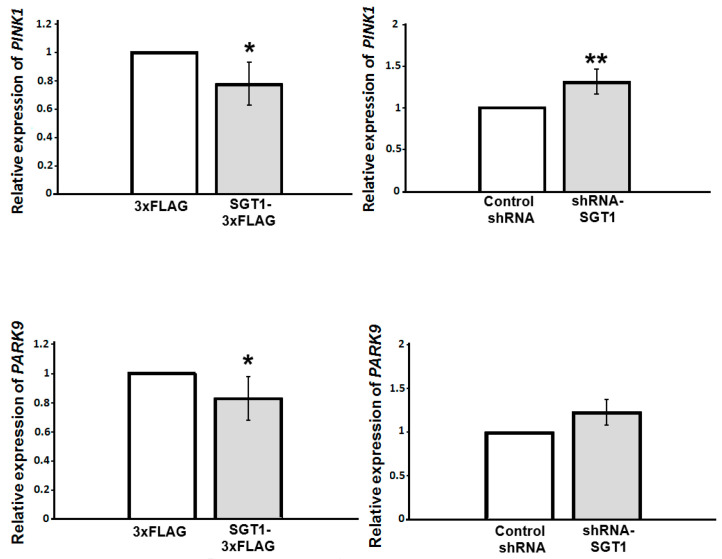
**Relative mRNA expression of *PINK1* (upper panels) and *PARK9* (lower panels) in HEK293 cells.** Left panels—control cells (3×FLAG) or cells overexpressing SGT1 (SGT1-3×FLAG) and right panels—control cells (control shRNA) or cells with diminished SGT1 level (shRNA-SGT1). Statistical analysis of the results obtained from three independent experiments. * *p* ≤ 0.05; ** *p* ≤ 0.01.

Previously, it was shown that nuclear α-synuclein is involved in transcriptional regulation of various genes [6,10]. Since SGT1 alters subcellular localization of α-synuclein we checked, by applying RT-qPCR, whether it influences expression of PD-related genes such as *PINK1* and *PARK9*. As it is shown in Figure 5, expression of these genes is lower in SGT1 overexpressing cells, while in those with silenced SGT1, the expression of *PARK9* remains the same and that of *PINK1* is higher than in control cells.

## 4. Discussion

In this work we have found that SGT1 protected HEK293 cells from rotenone-induced toxicity and observed that altered level of SGT1 had an effect on α-synuclein subcellular localization in HEK293 cells. More precisely, we found that in control cells the protein was predominantly localized in the nucleus while in cells with overexpressed SGT1 the majority of α-synuclein was present in the cytoplasm. Interestingly, SGT1 homolog, CacyBP/SIP, was also found to protect cells from rotenone-induced toxicity [17] but altered level of CacyBP/SIP had no effect on α-synuclein subcellular localization in HEK293 cells (not shown). α-Synuclein is predominantly a cytoplasmic protein, although some studies pointed to its nuclear localization [4,5,6]. Moreover, earlier results showed that in neuroblastoma SH-SY5Y cells and *Drosophila* brain cells nuclear α-synuclein was more toxic than cytoplasmic α-synuclein [4]. Thus, it is possible that the beneficial effect of SGT1 on rotenone-treated HEK293 cells may, at least in part, be due to its ability to influence cellular localization of α-synuclein. A positive result of the PLA experiment, which serves to detect molecules located in close proximity, suggests that SGT1 and α-synuclein likely co-exist in the same protein complexes (Figure S1). Therefore, it is conceivable that SGT1 may engage α-synuclein in cytoplasmic complexes and prevent its accumulation in the nucleus.

The results we have obtained show also that there is a correlation between the nuclear level of α-synuclein and expression of PD-related genes such as *PINK1* and *PARK9*. This is in agreement with other studies showing that nuclear α-synuclein caused alterations in gene expression. For instance, microarray analysis pointed to 15 genes that were up- and 30 that were down-regulated in neuroblastoma cells overexpressing α-synuclein [25]. Recent RNA-seq analysis revealed that in pluripotent stem cells with increased α-synuclein level there were 131 up- and 115 down-regulated genes when compared to control cells [26]. Additionally, some other studies have demonstrated that α-synuclein could regulate the expression of transcription factors such as CREB and NFAT [3]. In this work we have found that sequestering of α-synuclein to cytoplasm, caused by overexpression of SGT1, changed the expression of PD-related genes, *PINK1* and *PARK9.* Interestingly, in our previous study we have found that SGT1 level was higher in PD brain [15]. As it is known, *PINK1* regulates, directly or indirectly, many signaling pathways involved in PD pathogenesis including mitochondrial and Ca^2+^ homeostasis, protection against damage of misfolded proteins, autophagy/mitophagy and endoplasmic reticulum-mitochondria crosstalk [27]. In turn, *PARK9* encodes a lysosomal ATPase, the mutations and deficit of which have been linked to an early-onset form of PD. Interestingly, it was found that elevated expression of this ATPase reduced α-synuclein toxicity [28]. 

Altogether, based on the results presented in this work we suggest that SGT1-dependent changes in α-synuclein subcellular localization and *PINK1* and *PARK9* gene expression strongly argue for the involvement of SGT1 in the pathogenesis of PD, however, more studies are required to elucidate its role in signaling pathways leading to PD development.

## 5. Conclusions

In this work we show that SGT1 protects cells from the toxicity of rotenone, an agent that evokes behavioral and histopathological symptoms of PD. Moreover, we found that in control HEK293 cells α-synuclein is predominantly localized in the nucleus while in cells with elevated SGT1 level it is mainly present in the cytoplasm. Additionally, we discovered that altered SGT1 level, most probably through regulating α-synuclein partitioning between cytoplasm and nucleus, influences expression of PD related genes, *PINK1* and *PARK9.* Overall, our results point to SGT1 as an important factor that may be involved in the pathogenesis of PD.

## Figures and Tables

**Table 1:** List of plasmids used for transfection of HEK293 cells.

Plasmid	Description	Source/Reference
**pcDNA4-α-synuclein-3×FLAG**	plasmid for α-synuclein expression in eukaryotic cells with 3×FLAG tag	[17]
**p3×FLAG-CMV-10**	plasmid for protein expression in eukaryotic cells encoding 3×FLAG tag	Sigma-Aldrich
**p3×FLAG-CMV-10-SGT1A**	plasmid for SGT1A expression in eukaryotic cells with 3×FLAG tag	[20]
**p3×FLAG-CMV-10-SGT1B**	plasmid for SGT1B expression in eukaryotic cells with 3×FLAG tag	Present work
**pPURhU6-Luc4**	plasmid encoding control shRNA against *Renilla* Luciferase mRNA	Present work
**pPURhU6-SGT1**	plasmid encoding shRNA against SGT1 mRNA	Present work

**Table 2:** List of primers used in RT-qPCR experiments.

Gene	Forward	Reverse
***18S***	5′-CGCCGCTAGAGGTGAAATTC-3′	5′-TTGGCAAATGCTTTCGCTC-3′
***PINK1***	5′-TATGGAGCAGTCACTTACAGAAAATCC-3′	5′-GGTGAAGGCGCGGAGAA-3′
***PARK9***	5′-ATGATGGCTGGGATCCCTTT-3′	5′-AACTATCCTCTTTGTCTCTTATTTCGATAAC-3′

## Data Availability

The data presented in this study are available in the main text, figures, tables and supplementary material.

## Supplementary Materials

The following are available online at https://www.mdpi.com/article/10.3390/biom11111675/s1, Figure S1: Presence of complexes comprising SGT1 and α-synuclein visualized in HEK293 cells by PLA.

## Abbreviations

BSA: bovine serum albumin; CacyBP/SIP, Calcyclin Binding Protein/Siah-1 Interacting Protein; CBF3, centromere binding factor 3; DTT, dithiothreitol; EGTA, ethylene glycol-bis(β-aminoethyl ether)-*N*,*N*,*N*′,*N*′-tetraacetic acid; HRP, horseradish peroxidase; HSP, heat shock protein; MTS assay, assay to measure cell viability; NOD1, Nucleotide-binding oligomerization domain-containing protein 1; PBS, phosphate buffered saline; PBS-T, PBS containing 0.05% Tween 20; SGT1, Suppressor of G2 allele of SKP1; SCF, Skp-cullin-F-box; SDS, sodium dodecyl sulfate; TBS, tris-buffered saline; TBS-T, tris-buffered saline containing 0.05% Tween 20.
