# Supplementary material for: Sgt1 Regulates α-Synuclein Subcellular Localization and Expression of Parkinson’s Disease Related Genes, PINK1 and PARK9
Source: Biomolecules. 2021 Nov 11;11(11):1675. doi: 10.3390/biom11111675 (PMC8615725; doi:10.3390/biom11111675)
Supplement: Supplementary file 1 [file biomolecules-11-01675-s001.zip › biomolecules-1424628-supplementary.pdf]

biomolecules

MDPI

Sgt1 Regulates α-Synuclein Subcellular Localization and Expression of Parkinson's Disease-Related Genes, *PINK1* and *PARK9*

Anastasiia Bohush[1], Agnieszka Góral[1], Małgorzata Sierant[2], Barbara Nawrot[2], Wiesława Leśniak[1] and Anna Filipek[1],*

[1]Nencki Institute of Experimental Biology, Polish Academy of Sciences, 02-093 Warsaw, Poland
[2]Centre of Molecular and Macromolecular Studies, Polish Academy of Sciences, 90-363 Lodz, Poland
* Correspondence: Anna Filipek, Tel: +48-22-589 23 32; Fax: +48-22- 822 53 42 ; E-mail: a.filipek@nencki.edu.pl

Materials and methods

Proximity ligation assay (PLA)

To detect the SGT1-α-synuclein complexes in HEK293 cells the proximity ligation assay (PLA, In situ PLA Technology, Sigma-Aldrich, St. Louis, MO, USA) was applied. Cells, grown on poly-lysine coated glass coverslips were fixed with 3% (w/v) paraformaldehyde in buffer containing 60 mM PIPES, 25 mM HEPES, 5 mM EGTA, 4 mM $MgCl_2$, pH 7.0 (PHEM buffer) for 20 min at room temperature. Then, cells were washed with PBS, incubated with 50 mM NH_4Cl in PHEM buffer for 10 min at room temperature. After washing with PBS, cells were permeabilized for 4 min with 0.1% (v/v) Triton X-100 in PHEM buffer and washed again with PBS. All the following steps were performed according to the manufacturer's protocol using reagents (except for primary antibodies) and buffers provided by the manufacturer (Sigma-Aldrich, St. Louis, MO, USA). After blocking, the incubation with primary antibodies mouse anti-α-synuclein antibodies (Abcam, Cambridge, UK) and rabbit anti-SGT1 (BD Biosciences, San Jose, CA, USA), diluted 1:100, was conducted for 2 h at 37ºC in a humidity chamber. Then, incubation with anti-rabbit IgG PLUS and anti-mouse IgG MINUS PLA-probes diluted 1:5 was carried out for 1 h at 37ºC in a humidity chamber. Following the ligation and amplification steps, the coverslips were immobilized on microscopic slides with a mounting medium containing DAPI (VectaShield, Sigma-Aldrich, St. Louis, MO, USA). In the control experiment the ligation step was omitted or SGT1 protein was not overexpressed. Cells were analyzed under a confocal microscope (LSM 800, Carl Zeiss, Jena, Germany), equipped with a 63×oil objective, in the Laboratory of Imaging Tissue Structure and Function (Nencki Institute of Experimental Biology, Warsaw, Poland).

Results

By applying a PLA assay, we have checked whether SGT1 and α-synuclein can be found in the same protein complexes in HEK293 cells. As it is shown in **Figure S1**, the red dots reflecting close proximity of both proteins are clearly detectable when anti-SGT1 and anti-α-synuclein antibodies were applied **(upper panel**). Such dots are not clearly visible in control experiment in which ligase, a critical reagent in this assay, was omitted **(middle panel)** or cells without SGT1 overexpression were used **(lower panel).** Moreover, these dots are predominantly present in the cytoplasm. Thus, it might be argued that SGT1 can sequester α-synuclein in the cytoplasm and prevent its nuclear accumulation.

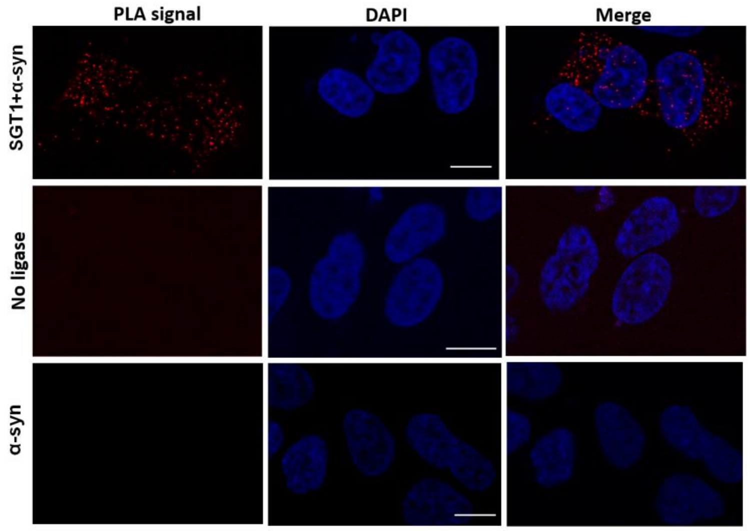

Figure S1. Presence of complexes comprising SGT1 and α-synuclein visualized in HEK293 cells by PLA. (Upper panel) Complexes of examined proteins are visible as red dots. **(Middle panel**) Control experiment in which ligase was omitted. **(Lower panel)** Control experiment in which cells without SGT1 overexpression were used. Cell nuclei, stained with DAPI, are in blue. Scale bar—10 μm.
